# Prevalence of body-focused repetitive behaviors in a diverse population sample – rates across age, gender, race and education – CORRIGENDUM

**DOI:** 10.1017/S0033291724002836

**Published:** 2024-11

**Authors:** Steffen Moritz, Jakob Scheunemann, Lena Jelinek, Danielle Penney, Stella Schmotz, Luca Hoyer, Dominik Grudzień, Adrianna Aleksandrowicz

The authors would like to make the following statement regarding the above article:

Our article is ambiguous as to whether the last column of Table 1 shows the current or lifetime prevalence of body-focused repetitive disorders (BFRDs). We would like to clarify that the current prevalence of BFRDs is displayed.
